# Supplementary material for: Predicting visual function by interpreting a neuronal wiring diagram
Source: Nature. 2024 Oct 2;634(8032):113–23. doi: 10.1038/s41586-024-07953-5 (PMC11446822; doi:10.1038/s41586-024-07953-5)
Supplement: Supplementary file 6 — CRF and ERF predictions for individual TmY4 and TmY9 cells. Analogous to Supplementary Data 3, but for TmY target types. Shown are the top four monosynaptic pathways, the strongest pathway passing through each of the top ten intermediary types (ranking from Extended Data Fig. 7), and the trisynaptic pathway Tm1–TmY–Dm3–TmY (see the section entitled Prediction of spatial normalization). [file 41586_2024_7953_MOESM6_ESM.zip › DataS4/TmY9q/720575940616170034.pdf]

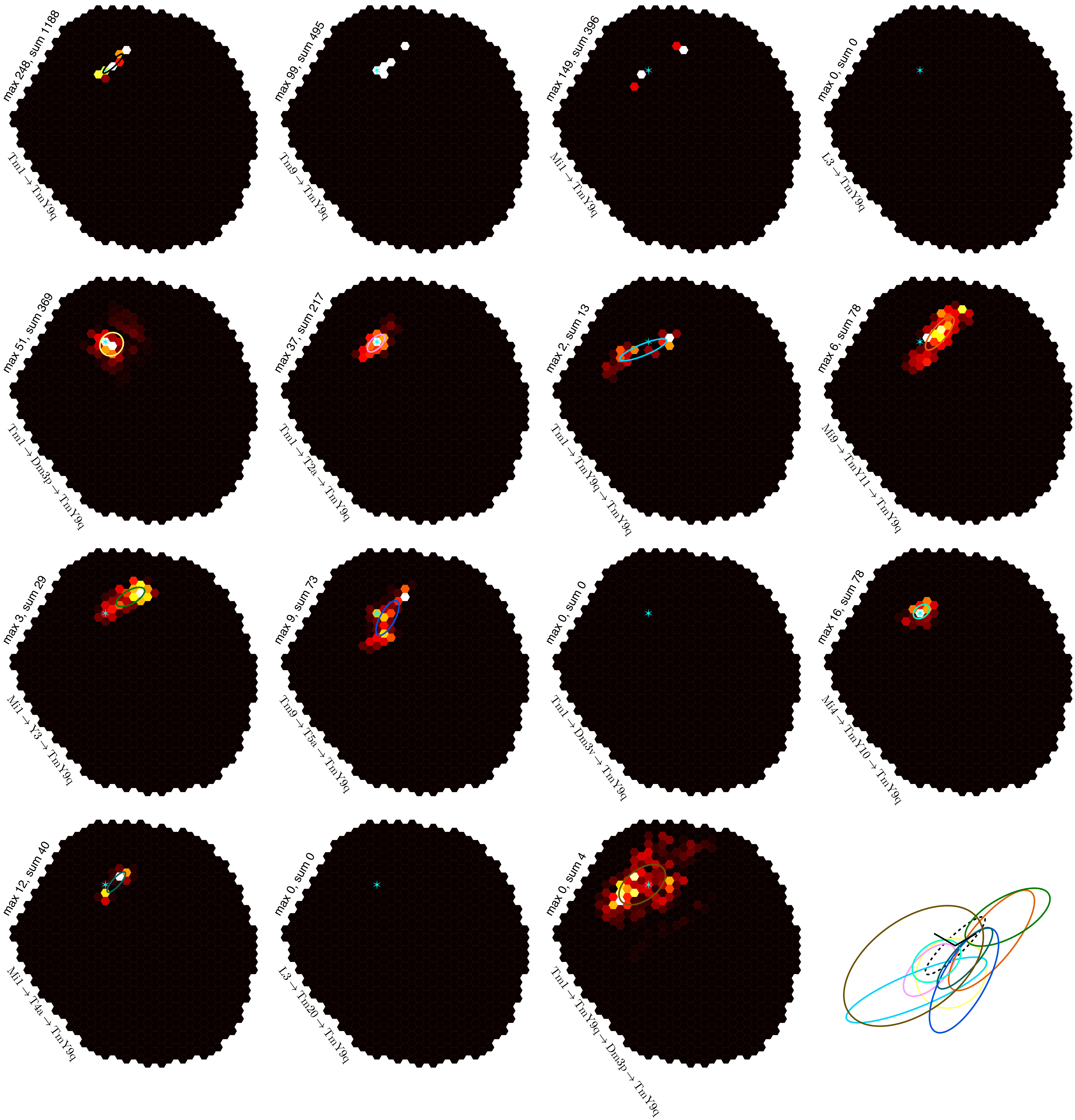
max 248, sum 1188
Tm1 → TmY9q
max 99, sum 495
Tm9 → TmY9q
max 149, sum 396
Mi1 → TmY9q
max 0, sum 0
L3 → TmY9q
max 51, sum 369
Tm1 → Dm3p → TmY9q
max 37, sum 217
Tm1 → T2a → TmY9q
max 2, sum 13
Tm1 → TmY9q → TmY9q
max 6, sum 78
Mi9 → TmY11 → TmY9q
max 3, sum 29
Mi1 → Y3 → TmY9q
max 9, sum 73
Tm9 → T5a → TmY9q
max 0, sum 0
Tm1 → Dm3v → TmY9q
max 16, sum 78
Mi4 → TmY10 → TmY9q
max 12, sum 40
Mi1 → T4a → TmY9q
max 0, sum 0
L3 → Tm20 → TmY9q
max 0, sum 4
Tm1 → TmY9q → Dm3p → TmY9q
